# The intraportal injection model: A practical animal model for hepatic metastases and tumor cell dissemination in human colon cancer

**DOI:** 10.1186/1471-2407-9-29

**Published:** 2009-01-24

**Authors:** Andreas Thalheimer, Christoph Otto, Marco Bueter, Bertram Illert, Stefan Gattenlohner, Martin Gasser, Detlef Meyer, Martin Fein, Christoph T Germer, Ana M Waaga-Gasser

**Affiliations:** 1 Department of Surgery, University of Wuerzburg Hospital, Wuerzburg, Germany; 2 Expertimental Surgery, Department of Surgery, University of Wuerzburg Hospital, Wuerzburg, Germany; 3 Department of Pathology, University of Wuerzburg Hospital, Wuerzburg, Germany; 4 Division of Surgery, Leopoldina Hospital, Schweinfurt, Germany

## Abstract

**Background:**

The development of new therapeutic strategies for treatment of metastasized colorectal carcinoma requires biologically relevant and adequate animal models that generate both reproducible metastasis and the dissemination of tumor cells in the form of so-called minimal residual disease (MRD), an expression of the systemic character of neoplastic disease.

**Methods:**

We injected immunoincompetent nude mice intraportally with different numbers (1 × 10^5^, 1 × 10^6 ^and 5 × 10^6 ^cells) of the human colon carcinoma cell lines HT-29 and SW-620 and investigated by histological studies and CK-20 RT-PCR the occurrence of hematogenous metastases and the dissemination of human tumor cells in bone marrow.

**Results:**

Only the injection of 1 × 10^6 ^cells of each colon carcinoma cell line produced acceptable perioperative mortality with reproducible induction of hepatic metastases in up to 89% of all animals. The injection of 1 × 10^6 ^cells also generated tumor cell dissemination in the bone marrow in up to 63% of animals with hepatic metastases.

**Conclusion:**

The present intraportal injection model in immunoincompetent nude mice represents a biologically relevant and adequate animal model for the induction of both reproducible hepatic metastasis and tumor cell dissemination in the bone marrow as a sign of MRD.

## Background

Colorectal carcinoma constitutes approximately 15% of all cases of cancer and is one of the most common malignant diseases worldwide. Despite multiple advances in its diagnosis and treatment, 20% to 45% of patients with colorectal carcinoma experience local recurrence and/or metastases with a consequent dramatic decline in prognosis. In the industrialized West, therefore, colorectal carcinoma is the second most common cause of death from cancer [1].

Metastases of colorectal carcinomas are initially localized in the liver in 40% to 80% of patients. The principal curative treatment option is surgical resection, although only one fourth of patients with liver metastases are operable [2]. Following initial radical curative surgery, metastases are attributed to the dissemination of tumor cells in the form of minimal residual disease (MRD), whose prognostic relevance continues to be discussed [3,4]. The classical view is that metastatic spread is a late process in malignant progression, but recent work suggested that dissemination of primary cancer cells to distant sites like the bone marrow might be an early event [5].

In recent years marked improvements have been made in the medicinal treatment of patients with colorectal carcinomas. New cytostatic agents and antibodies targeted against EGFR and VEGF have increased median survival in patients with advanced colorectal carcinomas to more than 2 years, almost doubling the survival time of the 5-FU era [6,7]. Reliable data are not available on the sequential application of available substances, an approach which could further increase survival.

Alternative chemotherapeutic and immunological treatment strategies are needed to further improve survival of patients with metastasized colorectal carcinoma. Biologically accurate animal models can play an important role in the development of such approaches and allow continued monitoring of their efficacy. For this purpose reproducible growth rates are needed with reliable formation of liver metastases and simultaneous induction and verification of MRD. Animal studies based on surgical orthotopic implantation are well capable of inducing liver metastases, but they are limited by highly variable growth kinetics and the need to prepare the tumor material in a subcutaneous (s.c.) tumor animal model [8,9]. For this reason numerous animal models involve injection of colon carcinoma tumor cells directly into the liver parenchyma, the spleen, or portal vein to induce tumor growth in the form of hepatic metastases of colorectal carcinoma [10-12]. It remains largely unclear, however, whether these animal models can induce the dissemination of tumor cells in the early stage of cancer progression, causing recurrence.

The aim of the present study was to use the intraportal injection of colorectal carcinoma cell lines in immunoincompetent mice to establish reproducible systemic metastases and dissemination of human carcinoma cells.

## Methods

### Animals

Six to 10-week-old female Balb/c nu/nu mice (Charles River GmbH, Sulzfeld, Germany) weighing 16–18 g at the time of surgery were used. The animals were kept in wire cages in laminar air flow cabinets at a temperature of 20–22°C, a humidity of 30–50%, and a 12-h/12-h light/dark cycle. Autoclaved chow pellets (Altromin GmbH, Lage, Germany) and autoclaved tap water were provided ad libitum. The animal colonies were maintained in a specific pathogen-free environment with regular screenings for the presence of murine pathogens (such as Mycoplasma pulmonis, Streptococcus pneumoniae, and Helicobacter spp.). Housing and all procedures involving animals were performed according to protocols approved by the University's animal care committee and in compliance with the guidelines on animal welfare of the National Committee for Animal Experiments.

### Cell lines

The human colon cancer cell lines HT-29 and SW-620 were propagated and cultured according to the distributor's protocol using McCoy's 5A medium modified and supplemented with 10% heat-inactivated FBS, streptomycin/penicillin (100 units/ml). Cells were cultured in a 5% CO_2 _incubator at 37°C, and the medium was changed every 3 days. Cultures were routinely tested for mycoplasma contamination to ensure that only negative cells were used.

### Surgical procedure

For intraportal injection of HT-29 and SW-620 cells the anesthetized animals were placed in a supine position. After disinfection of the skin in the area of surgery a median laparotomy was performed followed by mobilization of the duodenum to liberate the portal vein. Different numbers of tumor cells of both cell lines (1 × 10^5^, 1 × 10^6 ^and 5 × 10^6 ^cells) in 200 μl PBS solution were injected into the portal vein of 10 mice per group using a 32 G needle. After removal of the needle bleeding was stopped by gently pressing the puncture site with a commercially available cotton swab combined with a small piece of a styptic cellulose fleece. The injection procedure was considered successful if there was no post-injection bleeding from the puncture site or recoil of tumor cells within the injection canal or tumor cell spread into the abdominal cavity. After tumor cell injection the intestine was repositioned and the abdominal wall closed in a two-layer technique with non-absorbable sutures. Mice were sacrificed at 30 days post-injection or earlier if tumor related cachexy or hepatic failure with tumor related ascites occurred. Liver and lung tissue of all animals was collected and subjected to histological examination after sacrificing the animals by cervical dislocation. All surgical procedures were performed in accordance with institutional guidelines.

### Histology

Liver and the lung tissue of all animals were examined histological using standard hematoxylin and eosin staining. The histological material was assessed by a consultant pathologist.

### Isolation of mononuclear cells

To obtain tumor cells from bone marrow, both femoral bones of all animals were removed prior to dissection. The bone marrow was washed out with phosphate-buffered saline under sterile conditions. Mononuclear cell (MNC) fractions of the bone marrow were isolated by Ficoll (Pharmacia, Freiburg, Germany) density-gradient centrifugation at 400 *g *for 30 min. As a control, MNC of animals after intraportal injection of 0.9% saline were isolated in the same way (n = 5).

### Isolation of total RNA and cDNA synthesis

In all animals with hepatic metastases and in all control mice total RNA was extracted from 1 × 10^6 ^MNC with 1 ml Trizol reagent (Life Technologies GmbH, Karlsruhe, Germany) and cDNA synthesis was performed according to the manufacturer's recommendations (Applied Biosystems GmbH, Weiterstadt, Germany).

### CK-20 RT-PCR

The primers for human CK-20 are described by Weitz et al. [13]. They developed a nested PCR protocol with outer (first PCR) and nested (second PCR) primers. For the first PCR, 5 ul of the cDNA synthesis mix were used at a final volume of 50 μl 1× PCR buffer II containing 1.5 mM MgCl_2_, 5 μM (each) of sense and antisense outer primer, and 2.5 units of AmpliTaq DNA polymerase. The thermal cycling condition comprised an initial denaturing step at 95°C for 10 min and 35 cycles at 95°C for 30 sec, 60°C for 30 sec (annealing step), and 72°C for 30 sec (extension step) for 35 cycles, with a final extension step of 10 min. The second CK-20 PCR was performed in a manner similar to the first PCR. Five micro liters of the first PCR reaction were set up with nested primers at a final volume of 50 μl. Thirty-five cycles of amplification were performed at 30 sec intervals at temperatures of 95°C, 72°C, and 72°C. Glyseraldehyde-3-phosphate dehydrogenase (GAPDH) served as a control. The CK-20 primer pairs do not amplify murine CK-20 [14]. PCR products were analyzed by electrophoresis on 2% agarose gel.

## Results

### Procedure-related mortality

Severe portal vein bleeding following intraportal injection of tumor cells led to the death of one animal each after injection of 1 × 10^5 ^and 1 × 10^6 ^cells. Injection of 5 × 10^6 ^cells of the cell lines HT-29 and SW-620 was followed immediately by the death of one animal each, also due to massive portal vein bleeding. Noteworthy were the deaths within 24 hours of 2 animals after injection of 5 × 10^6 ^HT-29 cells and of 3 animals after injection of 5 × 10^6 ^SW-620 cells following an initially unproblematic procedure. At autopsy the cause of death was found to be large areas of infarction in the liver parenchyma that apparently led to acute liver failure. The overall injection-related mortality from the injection of 5 × 10^6 ^cells of both cell lines was 35%.

### Survival and tumor take rate

All animals surviving the injection of 1 × 10^5 ^cells of HT-29 and SW-620 free of complications also survived the 30-day study period and were sacrificed on the prearranged date. Of the animals receiving 1 × 10^6 ^cells, one died on the third day following injection of SW-620 cells. At autopsy no macroscopic bleeding or liver infarction were found, so the cause of death in this animal remains unexplained. All other animals in this injection group survived the 30-day study period unimpaired and were sacrificed on schedule.

The situation of the animals injected with 5 × 10^6 ^cells was entirely different. As mentioned above, 7 of 20 animals died during injection of the cell lines HT-29 or SW-620 or within 24 hours thereof. One other animal died of unknown causes on the fourth day post injection. On the 10th day following injection of 5 × 10^6 ^HT-29 cells, one animal had to be sacrificed due to severe tumor cachexy and swollen abdomen. Between the 10th and 20th days, 10 further animals exhibited such advanced tumor disease that they too had to be sacrificed. The sole surviving animal receiving 5 × 10^6 ^cells had to be sacrificed on the 25th day post injection, leaving not a single animal in this series to reach the scheduled endpoint of 30 days.

In animals injected with 1 × 10^5 ^cells, liver metastases occurred in 6 of 10 animals (60%) receiving HT-29 cells, in 5 of 9 (56%) receiving SW-620 cells. After injection of 1 × 10^6 ^cells, 8 of 9 animals (89%) in the HT-29 group and 7 of 9 animals (78%) in the SW-620 group had metastases in the liver parenchyma (Table). In both groups an average of 8 to 10, a few-mm-in-diameter, non-confluent liver metastases were detected in otherwise intact murine liver parenchyma. None of the animals were found to have pulmonary metastases.

**Figure 1:**
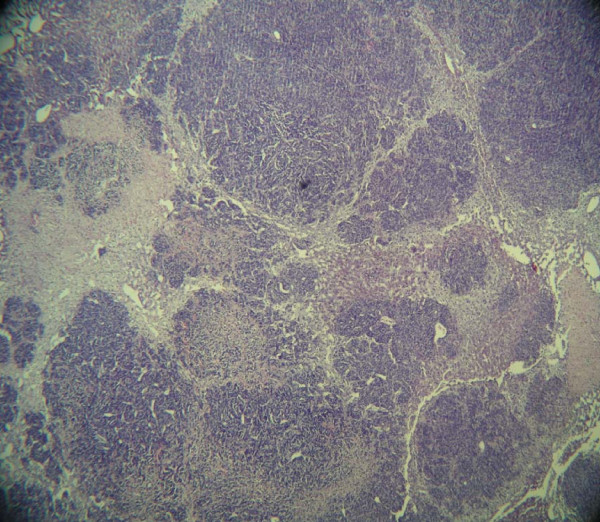
**Complete destruction of the liver parenchyma in a nude mouse on the 20^th ^day after intraportal injection of 5 × 10^6 ^cells of the colon carcinoma cell line HT-29**. Magnification: 40×, HE staining.

After injection of 5 × 10^6 ^cells of the HT-29 and SW-620 cell lines, all animals exhibited pronounced hepatic metastases with almost complete destruction of the liver parenchyma (Fig. 1). In the majority of animals, severe tumor disease with cachexy and swollen abdomen was seen between the 10th and 20th day after injection, none of these animals, as mentioned above, survived to term. In five animals each histologically confirmed pulmonary metastases were found after injection of 5 × 10^6 ^cells of the HT-29 and SW-620 cell lines.

**Figure 2:**
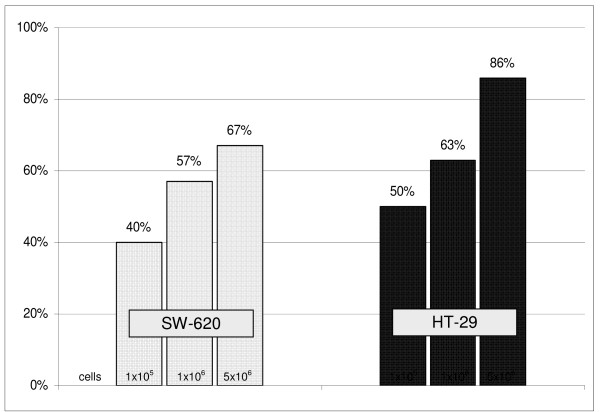
**Rate of CK 20 positivity in bone marrow indicating MRD following intraportal injection of different numbers of SW-620 and HT-29 cells in immunoincompetent mice**.

Twenty-four of the 39 animals (61.5%) with hepatic and/or pulmonary metastases were positive for CK-20 RT-PCR indicating dissemination of tumor cells in the bone marrow. Thus MRD was demonstrated in slightly less than two thirds of all animals with confirmed metastases after intraportal injection of HT-29 or SW-620 cells. With an increasing number of cells injected intraportally, the number of animals with CK-20 positive bone marrow also increased without reaching statistical differences in fisher's exact test (Fig. 2).

Bone marrow of nude mice was proved for the presence of disseminated tumor cells with a nested CK-20 RT-PCR in a gastric cancer model [14]. The sensitivity of the CK-20 RT-PCR was determined in dilution experiments as previously described [15]. It is possible to detect less than ten CK-20 positive tumor cells within 10^6 ^mononuclear cells. The total number of animals investigated, the procedure related mortality, the number with liver metastases, plus the number of animals with RT-PCR-confirmed MRD is shown in the table 1. A false positive signal was demonstrated in one of the non-cancer control animals of this study which is interpreted as a contamination since no CK-20 RT-PCR signals of non-cancer control animals could be detected in a different animal study (data not shown) demonstrating the high specificity of the primer pairs for human CK-20. In all RT-PCR experiments the internal control with murine GAPDH [14] showed a positive signal.

**Table 1:** Results of all intraportal injections with different cell counts of the colon carcinoma cell lines HT-29 and SW-620.

*Number of cells*	*Cell line*	*Total number of mice*	*Procedure related mortality (%)*	*Total number of mice after injection*	*Number of mice with liver metastases (%)*	*Number of mice with cytokeratin positivity in bone marrow (%)*
1 × 105	HT – 29	10	0%	10	60% (6/10)	50% (3/6)
	
	SW – 620	10	10%	9	56% (5/9)	40% (2/5)

1 × 106	HT – 29	10	10%	9	89% (8/9)	63% (5/8)
	
	SW – 620	10	0%	10	70% (7/10)	57% (4/7)

5 × 106	HT – 29	10	30%	7	100% (7/7)	86% (6/7)
	
	SW – 620	10	40%	6	100% (6/6)	67% (4/6)

## Discussion

A basic sign of unfavorable prognosis in colorectal carcinoma patients who have undergone curative resection is the occurrence of distant metastases, mainly in the liver. Another prognostically unfavorable factor in solid gastrointestinal neoplasm is the presence of so-called minimal residual disease (MRD), which is characterized in part by the dissemination of tumor cells in bone marrow. Disseminated tumor cells are held to be a possible cause of the recurrence of cancer [16,17]. It is therefore essential that animal models of human colorectal carcinomas simulate both hepatic metastases and MRD. The usefulness of such a model would be increased when the results can be reproduced within a relatively brief time period.

Although animal studies based on the orthotopic implantation of human colorectal carcinoma tissue do simulate the occurrence of liver metastases, they are limited by highly variable growth kinetics and the need to prepare tumor material in a s.c. tumor animal model [8,9,18]. The recently described orthotopic microinjection of various human colon carcinoma cell lines in nude mice showed a sufficient metastases rate for individual cell lines, the induction of MRD in this model, however, is unclear, as it is in other reported intraportal, intrasplenic and intrahepatic injection models [19].

Intraportal injection of human colon carcinoma cell lines directly simulates hematogenous tumor extension in patients with colorectal carcinomas. The injection of tumor cells into the spleen with subsequent splenectomy, a technically simpler procedure than direct intraportal injection, does also induce liver metastases reproducibly [20]. Nevertheless we think that intraportal injection of tumor cells mimic the natural metastatic process more closely than intrasplenic injection does even considering that intraportal injection of tumor cells circumvents the early steps in the genesis of metastasis in colorectal carcinoma. Although a rate of 10% portal venous thrombosis following intraportal injection is described [21] we did not observe any thrombotic complication in our series. Nevertheless this model has convincing advantages, since the metastases develop more rapidly than those in orthotopic xenotransplantation and thus avoid the non-T-cell-mediated rejection mechanisms of immunoincompetent animals, which are known to increase with increasing age of the animals [22].

A vital prerequisite for a practical animal model is acceptable perioperative mortality. In the present study this was achieved in part by the use of an operation microscope providing 16-fold magnification of the surgical field, which allowed sufficiently careful insertion of the injection needle into the 1.5 mm-in-diameter vessel lumen of the 16–18 g nude mice. If a 32-gauge needle is used, withdrawal of the injection cannula can lead to massive bleeding which led to the loss of 4 animals in the present study. We were able to control the bleeding by applying cellulose strips. Much more important for perioperative mortality than blood loss, however, was the injection of larger cell counts. Injection of 5 × 10^6 ^cells of each cell line was followed by 2 and 3 additional deaths within 24 hours after complication-free procedures. Autopsy revealed large, wedge-shaped, partially confluent areas of infarction that apparently led to acute liver failure in the affected animals. These may have resulted from the occlusion of large intraparenchymal liver vessels due to the presence of agglutinated tumor cells.

The injection of larger cell counts (5 × 10^6^) not only increased the perioperative mortality but also led to higher tumor-related mortality over the 30-day study period. By injecting 5 × 10^6 ^tumor cells of each of the 2 cell lines we achieved a metastases rate of 100%. Unfortunately, the rapid tumor growth led to such a rapid decline in their general condition that all of the animals had to be sacrificed prematurely. This situation certainly does not simulate a continually developing tumor as a biologically relevant animal model should. The high operation-related and tumor-related mortality associated with the injection of 5 × 10^6 ^tumor cells indicates that no more than 1 × 10^6 ^tumor cells should be injected in the intraportal injection model in nude mice.

In solid gastrointestinal neoplasm, the dissemination of tumor cells and/or MRD is regarded as a negative prognostic factor since they may cause tumor recurrence [17,23]. Considering the systemic character of solid tumors [24], MRD is characterized in part by the dissemination of tumor cells in bone marrow. The induction of MRD in an animal model therefore represents a highly promising tool for the development of specific therapeutic approaches.

## Conclusion

In the present study, CK-20-positive cells corresponding to disseminated tumor cells were detected in 24 of 39 animals with metastases following intraportal injection of tumor cells, representing a relative frequency of 61.5% of all animals irrespective of the injected cell counts. In the group injected with 1 × 10^6 ^tumor cells, MRD was found in about 60% of all animals with evident metastases. This tumor animal model therefore does simulate the induction of systemic tumor disease in animals with metastases. The fact that the differences in the rate of MRD following intraportal injection of different numbers of tumor cells did not reach statistical significance is due to the small number of animals investigated. But the trend is evident. The MRD rate in the present tumor animal model accords with that reported by another work group, which investigated MRD in a nude mouse model after orthotopic implantation of a high-grade malignant gastric carcinoma cell line [14].

Injection of human colon cancer cell lines in immunoincompetent nude mice led to reproducible hepatic metastases within a relatively short time period. The systemic character of the malignant disease induced by the tumor cell injection was confirmed for the first time by the demonstration of disseminated tumor cells in bone marrow. The present model is therefore well suited for the testing and establishing of tumor-specific therapeutic approaches.

## Competing interests

The authors declare that they have no competing interests.

## Authors' contributions

AT was responsible for all animal related procedures including the intraportal injection, participated in histological diagnosis and drafted the manuscript. CO was responsible for the molecular biological studies and participated in the preparation of the manuscript. MB participated in the animal studies and the surveillance of operated mice. BI established parts of the molecular biological studies and participated in data analysis. SG was responsible for the assessment of the histological material. MG participated in the animal studies and the surveillance of operated mice. DM conceived of the intraportal injection model and was involved in establishing microsurgical techniques. MF participated in the preparation of the manuscript. CTG and AMWG participated in the design of the study and its coordination. All authors read and approved the final manuscript.

## Pre-publication history

The pre-publication history for this paper can be accessed here:

http://www.biomedcentral.com/1471-2407/9/29/prepub
